# *In vivo* Functional Genomics for Undiagnosed Patients: The Impact of Small GTPases Signaling Dysregulation at Pan-Embryo Developmental Scale

**DOI:** 10.3389/fcell.2021.642235

**Published:** 2021-05-25

**Authors:** Antonella Lauri, Giulia Fasano, Martina Venditti, Bruno Dallapiccola, Marco Tartaglia

**Affiliations:** Genetics and Rare Diseases Research Division, Ospedale Pediatrico Bambino Gesù, IRCCS, Rome, Italy

**Keywords:** small GTPases, rare diseases, undiagnosed patients, next generation sequencing, *in vivo* models, zebrafish, functional genomics

## Abstract

While individually rare, disorders affecting development collectively represent a substantial clinical, psychological, and socioeconomic burden to patients, families, and society. Insights into the molecular mechanisms underlying these disorders are required to speed up diagnosis, improve counseling, and optimize management toward targeted therapies. Genome sequencing is now unveiling previously unexplored genetic variations in undiagnosed patients, which require functional validation and mechanistic understanding, particularly when dealing with novel nosologic entities. Functional perturbations of key regulators acting on signals’ intersections of evolutionarily conserved pathways in these pathological conditions hinder the fine balance between various developmental inputs governing morphogenesis and homeostasis. However, the distinct mechanisms by which these hubs orchestrate pathways to ensure the developmental coordinates are poorly understood. Integrative functional genomics implementing quantitative *in vivo* models of embryogenesis with subcellular precision in whole organisms contribute to answering these questions. Here, we review the current knowledge on genes and mechanisms critically involved in developmental syndromes and pediatric cancers, revealed by genomic sequencing and *in vivo* models such as insects, worms and fish. We focus on the monomeric GTPases of the RAS superfamily and their influence on crucial developmental signals and processes. We next discuss the effectiveness of exponentially growing functional assays employing tractable models to identify regulatory crossroads. Unprecedented sophistications are now possible in zebrafish, i.e., genome editing with single-nucleotide precision, nanoimaging, highly resolved recording of multiple small molecules activity, and simultaneous monitoring of brain circuits and complex behavioral response. These assets permit accurate real-time reporting of dynamic small GTPases-controlled processes in entire organisms, owning the potential to tackle rare disease mechanisms.

## Introduction

Rare diseases are individually uncommon but collectively frequent, affecting approximately 25 million people in Europe and impacting between 263 million and 446 million people worldwide (Wakap et al., 2020), with a significant proportion of cases awaiting diagnosis (sources: Orphanet, Eurordis, and WHO, Kaplan et al., 2013). They are often chronic, degenerative, and disabling conditions, which in approximately 70% of cases have a pediatric onset and show high morbidity and mortality. As estimated by the BURQOL-RD project (“Social Economic Burden and Health-Related Quality of Life in Patients with Rare Diseases in Europe”), a high level of socioeconomic burden is associated with these conditions (Angelis et al., 2015), which challenges health care systems globally, as well as the quality of life of the patients and their families. Particularly dramatic is the situation for pediatric cancers, which, despite their rarity, represent a significant disease burden nowadays. Yearly, more than 500,000 new cases of rare cancers are diagnosed (Gatta et al., 2017), causing approximately 6000 deaths in children, according to the European Society for Pediatric Oncology. The World Health Organization estimates that half of these tumors are malignant hematological cancers (e.g., leukemia) or solid nervous system tumors (e.g., neuroblastoma) (Gupta et al., 2015; Steliarova-Foucher et al., 2017).

A significant proportion of these disorders underlie one or more genetic alterations causing functional dysregulation of master regulators involved at various levels and stages of complex and dynamic developmental programs (e.g., cell proliferation, migration, differentiation, and developmental competence) of virtually any growing tissue or organ. Molecularly, a relatively small number of signaling pathways and networks (Wingless/Integrated (Wnt), hedgehog (Hh), Notch, Bone morphogenetic protein (BMP), fibroblast growth factor (FGF), mitogen-activated protein kinase/extracellular signal-regulated kinase (MAPK/ERK), etc.) are responsible for directing developmental programs. The crosstalk among these pathways, together with positive and negative control loop stations mediated by highly conserved molecular nodes, accounts for the pleiotropy of signaling, which ultimately shapes organismal development. These pathways’ interplays ensure differential responses to converging – and sometimes conflicting – messages and thereby multiorgan morphogenesis and homeostasis (Basson, 2012). Therefore, it is not surprising that from various alterations of core signaling hubs mastering multiple developmental networks, both developmental syndromes and malignancies arise. Yet, our biological knowledge on the key genes, the regulated signaling pathways, and the intracellular nodes differentially involved in development and disease remains poor. The current lack of a case-specific mechanistic understanding further hinders the disease identification, leaving many of them “orphan” of an accurate “diagnosis” and therefore targeted cure. This knowledge gap is particularly challenging, given the short life expectancy associated with a large fraction of rare conditions (Courbier and Berjonneau, 2017).

Following the EU call for action, revolutionary sophistication and rapid implementation of next-generation sequencing (NGS) techniques, especially whole-exome sequencing (WES), have allowed a considerable boost in the identification of genomic modifications and signaling pathways’ alterations in the field of rare diseases. Recently, the National Institutes of Health (NIH)–supported Centers for Mendelian Genomics noted an unprecedented increase in the number of novel diseases discovered per year, estimated to be more than 200 (Posey et al., 2019). Clearly, besides identifying the genes, precise fingerprints of disease mechanisms would help create a new “taxonomy of the disease” with immediate benefit on patient care specialization. Yet, for many of the newly discovered genetic conditions, even the physiological activity of the proteins involved remains poorly known. To resolve this gap, it is beneficial to invest into the smart combination between (i) *in silico* wide genome search for disease–gene/pathogenic variants in undiagnosed patients enrolled in international networks and (ii) functional genomics approaches using *ad hoc in vitro* systems (i.e., iPS, patient-specific induced pluripotent stem cells), supported and enhanced by (iii) both vertebrate and invertebrate animal disease models. Along this line, in the past decade, others and we have contributed to decisive advancements in the understanding of the pathophysiological role of a number of small GTPases belonging to the large RAS superfamily. In an international research framework dedicated to undiagnosed patients started at the “Ospedale Pediatrico Bambino Gesù” children’s hospital, functional genomic studies employing WES analysis and complementary *in vitro* experimental approaches, as well as invertebrate and vertebrate models, have allowed us to identify new nosologic entities caused by mutations affecting several genes, including a subset of which encode small GTPases. For instance, we associated a uniquely behaving genetic alteration in *CDC42* [OMIM: 116952] with a severe autoinflammatory condition (Lam et al., 2019). The specific molecular profiling of these patients allowed prompt lifesaving treatment, whereas validation of the pathogenicity in nematodes and human immune cells unraveled the impact on development, hematological cell maturation, and motility (Lam et al., 2019). Of note, we previously identified a different class of mutations affecting the same genes as the cause of a clinically variable neurodevelopmental disorder (Martinelli et al., 2018), emphasizing the requirement of functional characterization analyses to casually associate genomic variants with disease and decipher the underlying mechanisms. More recently, we identified activating mutations in the gene encoding the key effector of the MAPK signaling cascade, *MAPK1* [OMIM: 176948], as cause of a neurodevelopmental disease within the RASopathies spectrum (Motta et al., 2020). Again, *in vivo* assays in the context of cell differentiation and morphogenesis contributed to the validation of the pathogenicity of the mutations during embryonic development. The work provided evidence for a differential impact of germline inherited (found in developmental disorders) and somatically acquired (cancer-associated) mutations in this gene. The expansion of such paradigm in modern biomedical research clearly represents a valid tool for deepening our understanding of healthy and diseased mechanisms as well as core developmental hubs.

Here, we review recent functional genomics findings proving mutations in some among the large group of small GTPases molecules to be critically involved in rare developmental syndromes and cancers. We next discuss the current knowledge on the interplay with signaling pathways networks, whose tightly regulated activity is essential in many developmental processes, from lateral inhibition (which differentiates cell fate from initial equivalence fields) to cell polarity mechanisms (instructing gastrulation cell movements) and is being implicated in pediatric diseases. The cost reductions in sequencing and the extraordinary progress in functional imaging, signal biosensors optimization, and genome engineering in living organisms are opening long-awaited possibilities to combine in a single workflow (a) analyses directed to identify new disease genes with (b) sophisticated functional approaches *in vivo* to validate the putative pathogenic variants. Global conservation of the genes exists across taxa such that smartly chosen tractable model systems and *ad hoc in vivo* tools are and will be crucial for the majority of the newly discovered diseases for which we still fail to understand the impact on the signaling networks during development. In this context, we briefly discuss the advantages in using zebrafish for functional genomics of rare diseases and examine the latest tools, which enable highly resolved *in vivo* whole-embryo real-time reporting of dynamic small GTPase-regulated processes during development.

## The RAS Superfamily of Small GTPases in Development and Disease

**FIGURE 1:**
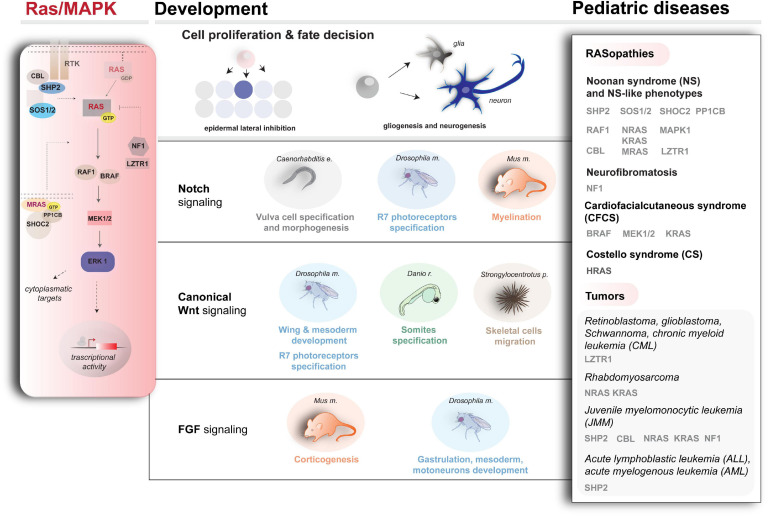
Schematic representation of Ras/MAPK cascade (left), Ras/MAPK-influenced pathways, and developmental processes (center) and examples of genetic conditions underlying a dysregulated cascade (right). For the diseases and disease–genes depicted here, besides the literature cited in the text, refer to Roberts et al. (2007) and Tartaglia et al. (2007) (*SOS1* mutations in NS), Carta et al. (2006); Pandit et al. (2007); Cordeddu et al. (2009); Cirstea et al. (2010) (*KRAS*, *NRAS*, *RAF1*, and *SHOC2* mutations in NS and related conditions), Aoki et al. (2005, 2013) (*HRAS* mutations in CS), Flex et al. (2014) (*RRAS* mutations in a RASopathy condition prone to cancer), Yamamoto et al. (2015) (*SOS2*, *LZTR1* mutations in NS), Martinelli et al. (2010, 2015); Pérez et al., 2010 (*CBL* mutations in a developmental syndrome prone to cancer), Urosevic et al. (2011); Aoidi et al. (2018) (*BRAF*, *MEK1*, or *MEK2* mutations in CFCS), and Capri et al. (2019) (*RRAS2* mutations in NS).

**FIGURE 2:**
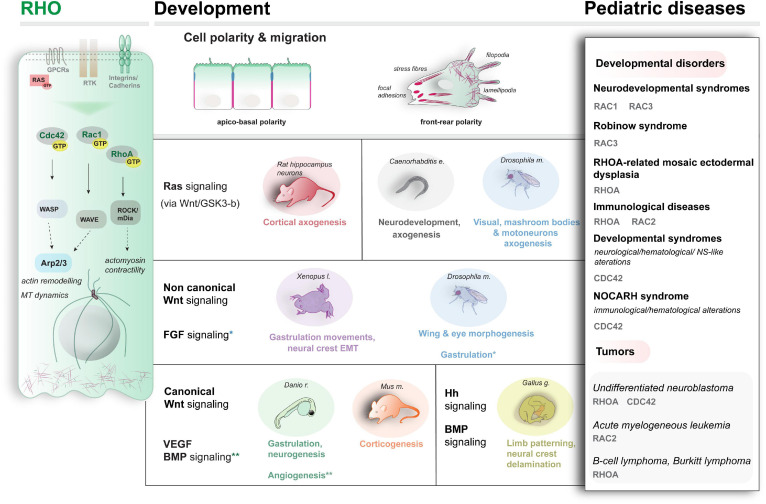
Schematic representation of the main RHO proteins’ activity (left), examples of the developmental pathways and processes modulated by RHO (center) and examples of genetic conditions associated with altered Rho activity (right).

**FIGURE 3:**
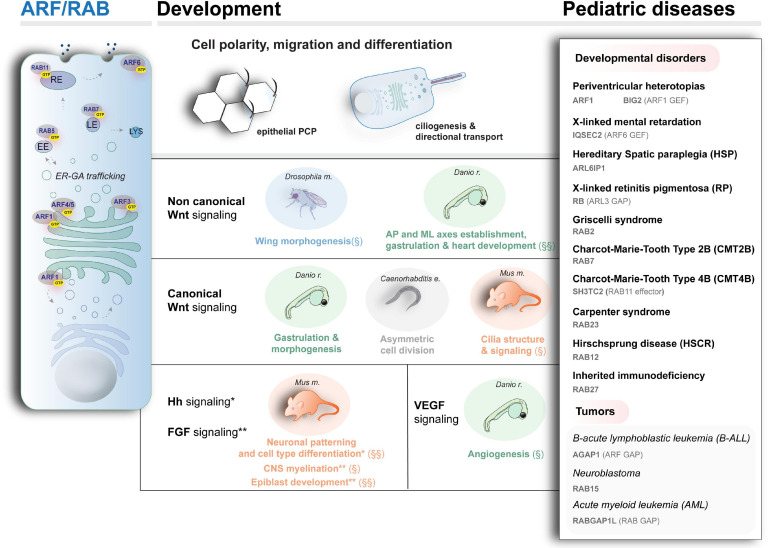
Schematic representation of intracellular trafficking and organelles associated with ARF and RAB activity (left), the main developmental pathways and processes modulated by vesicular trafficking (center), and examples of rare diseases caused by mutant ARF and RAB proteins. § and §§ indicate processes associated with ARF and RAB proteins, respectively. No symbol indicates evidence for processes involving both ARF and RAB activity. EE, LE, RE: early, late, and recycling endosomes, respectively. LYS: lysosomes. For the disease genes and diseases depicted here, besides the literature cited in the text, refer to Novarino et al. (2014); Wakil et al. (2019) (*ARF-like 6 interacting protein 1* mutations in neuropathy with spastic paraplegia, microcephaly, leukoencephalopathy, and seizures); Griscelli and Prunieras (1978) (mutations in *RAB2* in Griscelli syndrome affecting the immune system); Cogli et al. (2009); Lupo et al. (2009); Stendel et al. (2010), for the motor and sensory neuropathies Charcot–Marie–Tooth type 2B and type 4 (CMT2B and CMT4), caused by mutations in RAB7 and RAB11 effector SH3TC2, respectively; Harvey et al. (2010) (mutations in the ARF-specific GAP encoding gene *AGAP1* in pediatric high-risk B-cell ALL); Roberti et al. (2009) (gene rearrangement in the RAB-specific GAP encoding gene *RABGAP1L* in patient with Klinefelter syndrome who developed AML).

The RAS superfamily of small GTPases comprises five main protein families grouped by structure and function. They include proteins belonging to the (i) RAS family, involved in cell proliferation, specification, and differentiation (Figure 1); (ii) RHO family, known to influence actin and microtubule (MT) cytoskeleton and thereby cell migration and morphology (Figure 2); (iii) RAN family, which control nuclear transport (Figure 3); and (iv) ADP-ribosylation factor (ARF) and RAB families, involved in various steps of vesicle trafficking and organelles’ dynamics (Wennerberg et al., 2005). They all regulate their activity by cycling between an active (GTP-bound) and an inactive (GDP-bound) form, a switch determined by a number of extracellular signals and effectors (Bourne et al., 1991). By this dynamic activity and the myriad of effectors, these small GTPases function as molecular hubs at the crossroad between morphogenetic inputs, crucial for signal integration to determine cell precursors’ state, behavior, and specification in developing organisms. Given the plethora of cell processes that they assist, these proteins and their related signaling components have long emerged as major players in developmental disorders and malignancies (Schubbert et al., 2007; Simanshu et al., 2017; Qu et al., 2019; Figures 1–3). Indeed, the list of disease-causing mutations affecting signalings modulated by proteins of the RAS superfamily is increasing. However, we only begin to understand the complexity of their role in developmental pathways and their relevance for the onset of disease. Here, we focus on the emerging rare disease–causing genes encoding proteins of the RAS, RHO, ARF, and RAB families and the known mechanistic consequences altering developmental processes and relevant for pathogenesis, also emerging from *in vivo* models.

## RAS/MAPK Signaling Cascade and Its Dysregulation in Developmental Disorders and Cancer

Since the identification of the RAS proteins in the 1980s, the biochemistry of their signaling and their role as regulators of multiple cellular processes (e.g., proliferation, survival, differentiation, and apoptosis) in development and homeostasis has been intensively examined (Downward, 1998; Drosten et al., 2010; Kang and Lee, 2019). Various growth factors, cytokines, and hormones activate the Ras signaling network leading to the MAPK cascade and other pathways (Schlessinger, 2000) in a highly conserved manner (Rojas et al., 2012). A schematic overview of the Ras/MAPK signaling pathway is shown in Figure 1, left. Briefly, autophosphorylation of cell surface receptor tyrosine kinases promotes membrane recruitment of guanine nucleotide exchange factors (GEFs, e.g., SOS). This triggers the binding of RAS to GTP, activating the signaling (Cox and Der, 2010). The phosphatase SHP2 further contributes to RAS activation by inactivating regulatory tyrosines in receptors and scaffolding proteins (Matozaki et al., 2009). On the other hand, deactivation of RAS is promoted by GTPase-activating proteins (GAPs), such as neurofibromin 1 (NF1), via positive regulation of GTP hydrolysis (Vigil et al., 2010) or via ubiquitination directly by a recently identified cullin3-RING ubiquitin ligase complex (Steklov et al., 2018; Motta et al., 2019; Abe et al., 2020) and via the E3 ubiquitin ligase CBL by functional downmodulation of the activated cell surface receptors (Mohapatra et al., 2013). Downstream kinases (e.g., RAF1) are responsible for translating Ras signaling into the activation of MAPK ERK kinases (MEKs), resulting in the activation of ERKs (extracellular signal–regulated kinases), which phosphorylate various cytoplasmic and nuclear targets to mediate cellular responses. A phosphatase complex (MRAS, SHOC2, and PP1CB) dephosphorylates a single inhibitory site on RAF kinase, activating signal flow through the MAPK cascade. Depending on the cellular context, the strength and length of signaling, proliferation, apoptotic, or differentiation signals can be triggered (Downward, 1998; Murphy et al., 2004; Drosten et al., 2010; Kang and Lee, 2019).

Considering the plethora of functions of Ras/MAPK signaling during development, it is clear that mutations affecting signaling backbone’s core components have various deleterious effects in terms of development. Indeed, germline mutations affecting different components of signaling cascade are responsible for RASopathies, a group of developmental disorders comprising Noonan syndrome (NS), LEOPARD syndrome (NS with multiple lentigines), Costello syndrome (CS), cardiofaciocutaneous syndrome (CFCS), neurofibromatosis type 1 (NF-1), and other clinically related disorders, displaying high genetic and clinical heterogeneity (for a comprehensive review of the work in the field, refer to Cox and Der, 2010; Tartaglia et al., 2011; Rauen, 2013; Simanshu et al., 2017; Tajan et al., 2018; and more recently Kang and Lee, 2019). It is also equally established that activating mutations in genes encoding members of the Ras/MAPK signaling are commonly associated with cancers (Malumbres and Barbacid, 2003). They are among the primary causes of several pediatric malignancies affecting the nervous system, such as gliomas and astrocytomas, which show the highest degree of mortality in children. Myeloproliferative syndromes, such as juvenile myelomonocytic leukemia (JMML) and pediatric acute lymphoblastic leukemia (ALL) are also characterized by hyperactivated Ras/MAPK signaling (Zhang et al., 2011; Rauen, 2013). Of note, pediatric patients affected by NS, CS and NF show increased cancer predisposition (Brems et al., 2009; Kratz et al., 2015) with a high incidence of leukemia (Emanuel, 2004; Strullu et al., 2014) and other cancers [i.e., neuroblastoma or rhabdomyosarcoma (RMS); Moschovi et al., 2007].

After the discovery of germline gain-of-function mutations in *PTPN11* (encoding SHP2), the first gene involved in NS and LS (Tartaglia et al., 2001; Digilio et al., 2002; Legius et al., 2002), and of somatic activating mutations in contributing to JMML and acute leukemia (Tartaglia et al., 2003; Jongmans et al., 2011), enormous work was carried out via NGS, which disclosed a plethora of new disease-causing genes and mutations. Combined with increasingly sophisticated functional investigations, this approach is contribuiting to depict a complex mechanism of action of Ras/MAPK signaling in the pathophysiology of diseases (Kang and Lee, 2019). Besides the cases discussed here, an overview of the main members of Ras/MAPK signaling for which mutations have been described over the years that associate with developmental diseases and malignancies is shown in Figure 1 (right). For clinical and genetic review, refer to Tartaglia and Gelb (2010); Tidyman and Rauen (2016); Tajan et al. (2018). For mechanistic investigations, several animal models are available, which recapitulate various aspects of RASopathies (Jindal et al., 2015; Tajan et al., 2018). Among these, NS mice models showcase the impact of *PTPN11* mutations on neuronal and glial cell development (Araki et al., 2004; Gauthier et al., 2007; Ehrman et al., 2014), whereas in zebrafish an *NRAS*-depending NS phenotype rescued by MEK inhibitors was successfully modeled (Runtuwene et al., 2011). Moreover, besides rodents (Schuhmacher et al., 2008), fish models for CS caused by hyperactive HRAS*-*G12V variant are also available, which recapitulate the human condition and associate with tumorigenesis (Santoriello et al., 2009).

### Recent Genetic Findings in Rasopathies and Pediatric Tumors

A large repertoire of genetic conditions described since the discovery of *PTPN11* mutations, and more genes and mutations linked to the Ras/MAPK cascade and impacting developmental programs as well as cancer onset continue to be characterized by NGS and functional genomics efforts (Figure 1, right). Among the recent findings, loss of function and dominant negative mutations in *LZTR1*, which disables the ubiquitination of RAS and thereby the suppression of the signaling, were found in NS and pediatric cancers (Bigenzahn et al., 2018; Steklov et al., 2018; Motta et al., 2019; Abe et al., 2020). Noteworthy, Gröbner et al. (2018) have recently established *LZTR1* mutations as a hereditary factor predisposing to pediatric retinoblastoma, hypodiploid B-cell ALL, and high-grade glioma K27wt. Inactivating mutations of *LZTR1* have also been associated with drug resistance in RAS-induced chronic myeloid leukemia (Bigenzahn et al., 2018), whereas another class of both loss of function and dominantly acting *LZTR1* mutations seems to predispose to the development of glioblastoma and adult-onset schwannomatosis (a rare cancer-prone disorder) (Piotrowski et al., 2014; Paganini et al., 2015; Motta et al., 2019).

Of note, *Drosophila* and murine models exist for this condition, which showed the involvement in morphogenesis (Bigenzahn et al., 2018) and in Schwann cells’ behavior to shift from quiescent supporting cells into a highly dedifferentiated and proliferating state (Steklov et al., 2018). The recent establishment of vital CRISPR/CAS-based zebrafish *lztr1* null models expands further the possible comparative work (Nakagama et al., 2020).

Lastly, WES sequencing in a group of patients showing neurodevelopmental alterations within the RASopathy spectrum coupled to functional validation in nematodes has more recently established the pathogenicity of *de novo* mutations affecting MAPK1 (ERK2) directly and possibly their ability to interact with regulators and effectors (Motta et al., 2020). The underlying mechanism and plausible perturbance of fine signaling balances within developmental programs remain to be characterized.

## Functional Relevance of Developmental Signalings’ Interplay Involving RAS/MAPK for Cell-Type Specification, Rasopathies, and Pediatric Tumors

The differential impact of the Ras/MAPK signaling during embryogenesis likely depends largely on the cross-modulatory signaling interplays in which RAS activity is involved, which contribute to determine combinatorial codes that stir developmental competencies into specific cell fate (Halfon et al., 2000). A schematic overview of some of the main developmental signalings and processes involving the Ras/MAPK pathway is shown in Figure 1 (center). Functional studies in convenient model systems (such as the developing vulva in nematodes, the compound eye in insects, the somites’ development in zebrafish and the rodent brain) are contributing to tease apart some of these interplays with relevant developmental pathways such as Notch (Sundaram, 2005) and Wnt (Jeong et al., 2018) and others, as shown by the examples below.

### Notch Signaling

Compelling evidence demonstrates that Ras/MAPK signaling is able to modulate Notch pathway both positively and negatively in various embryonic precursor fields, contributing to the critical balance between inductive and inhibitory inputs, which instruct cell-type specification within a single equivalence domain. For instance, this mechanism is instrumental in generating lateral inhibition in progenitor cells during vulva patterning in developing *Caenorhabditis elegans* (Levitan and Greenwald, 1998; Chen and Greenwald, 2004; Sternberg, 2005).

The sequential interplay and various modes of crosstalk between Ras/MAPK and Notch inputs influence also progenitor specification during the development of prospective R photoreceptor cells (Tsuda et al., 2002; Roignant and Treisman, 2009), wing (Marygold et al., 2011), muscle and cardiac tissue in *Drosophila* (Carmena et al., 2002). Specifically, the activity of epidermal growth factor (EGF)-triggered Ras/MAPK cascade induces photoreceptor identity in the developing eye while promoting the expression of the Notch ligand Delta in the same cells, via the control of the transcriptional corepressor complex Ebi (transducin β-like 1, TBL1, in mammals). This mechanism based on inhibitory and inductive signals contributes to the acquisition of non-neuronal identity by neighboring cells and therefore to the global functional patterning of the differentiating cell clusters within the developing compound eye (Tsuda et al., 2002; Roignant and Treisman, 2009).

Moreover, in *Drosophila* wing and zebrafish somites’ development, EGF-dependent Ras/MAPK signaling induces reduction of the repressor activity of Groucho (Gro) to downmodulate Notch-controlled transcriptional output and likely that of other developmental pathways (Hasson et al., 2005).

A mammalian example is offered by rodent brain development. Here the establishment of cell identity is normally influenced by Ras/MAPK signaling, which contributes to balance neuronal and glial differentiation (elegantly summarized by Kang and Lee, 2019), regulating directly the expression of distinct proneural genes (e.g., *Neurog2* or *Ascl1*) during corticogenesis (Li et al., 2014). In this developmental context, a correct interplay between Ras/MAPK and Notch is likely crucial for myelinogenesis and relevant for pathology. Indeed, patients affected by NF-1, caused by dominant inactivating mutations in *NF1* resulting in an increase the active GTP-bound RAS (Cawthon et al., 1990; Wallace et al., 1990), show pronounced myelin damage, and decompaction is observed, as well a predisposition to life-threatening tumors (Brems et al., 2009; Ratner and Miller, 2015). Accordingly, recent *in vivo* experiments in mice demonstrated that NF1 loss of function, resulting in sustained Ras/MAPK signaling, causes myelin defects underlying a deregulation of Notch activity (López-Juárez et al., 2017).

On the other hand, an extensive collection of studies indicates the importance of a complex pathway interplay involving Ras/MAPK and Notch also in tumorigenesis (Fitzgerald et al., 2000). For instance, in neuroblastoma, which represents at least 8% of all pediatric cancers (Colon and Chung, 2011), transforming growth factor β–induced Ras signaling positively regulates the Notch pathway (Stockhausen et al., 2005). Mechanistically, work in rodents traced back RAS and Notch’s activity during early development of *nestin* + glial cells, which, if dysregulated, might trigger cancerogenic lesions at the level of the subventricular zone in gliomas (Shih and Holland, 2006). Lastly, rodent models also offered evidence that a mild hyperactivation of Notch1, behind the dose required for normal T-cell differentiation during development, contributes to leukemia onset, synergically with RAS activation (Chiang et al., 2008).

### Wnt Signaling

Evidence for a modulatory function of Ras/MAPK on Wnt in development is available from various animal models. Different modes of crosstalk between these two signalings have been described that contribute to adequate cellular response in different developmental contexts and timings. In the insect imaginal disk of the developing wing, for instance, tissue patterning is controlled via a conserved MAPK cascade downstream insulin-like growth factor, which regulates canonical Wnt pathway by stabilizing the β-catenin effector, thanks to a direct interaction with the ortholog of MEK1/2 (Hall and Verheyen, 2015).

Offering another type of example of the impact of cross-modulatory activity between Ras/MAPK and Wnt signaling, experiments in the invertebrate sea urchin suggested that maternally deposited β-catenin drives transcription of MEK and of the RAS target Ets1 during ectodermal-to-mesenchymal transition (EMT) in migrating skeletal precursor cells (Rottinger et al., 2004).

On the other hand, as suggested by zebrafish disease models, Ras/MAPK pathway seems to mediate the activity also of non-canonical Wnt signaling during vertebrate gastrulation (Kilian et al., 2003; Jopling et al., 2007). As an example, overexpression of *shp2* mutant variants in zebrafish embryos, recapitulating human NS and LS traits, indicates a Wnt-dependent effect of Shp2 on embryonic convergence and extension (CE) movements, resembling phenotypes found by downregulating the non-canonical Wnt (Kilian et al., 2003; Jopling et al., 2007). Zebrafish work further linked Wnt-dependent Shp2 activity even to RhoA signaling (Jopling et al., 2007), similarly to an interplay described already in frog development (O’Reilly et al., 2000). Of note, the activity of SHP2 seems to be crucial to influence also other signaling pathways relevant to developmental diseases and tumorigenesis, such as Hippo and Shh (Huang et al., 2014).

Moreover, *Drosophila* mesoderm specification shows a good example of a rather complex cross-modulation of the Ras/MAPK cascade on Wnt pathway for signal integration. In *eve* + domain, the muscle prepatterning signaling of the Wnt and TGF-β orthologs induces activation of Tin and Twi, which function together with Ras-activated Ets protein in tissue-specific enhancer domains to establish muscle and cardiac identity (Halfon et al., 2000).

Further manifesting the complexity of the interplays and multilevel integration of these signalings during development, the insect PDZ domain-containing protein called Canoe (Cno) mediates the crosstalk between Ras-, Notch-, and Wnt-induced pathways via interacting with disheveled (Dsh/Dvl). This Wnt-dependent mechanism seems to facilitate Ras induction and Notch signaling to finely modulate their relative signal intensities throughout mesoderm specification (Carmena et al., 2006). Similar interactions to induce R7 photoreceptors were observed in the developing compound eye (Cooper and Bray, 2000). Demonstrating the relevance of this crosstalk for pathology, in severe forms of acute myeloid leukemia (AML), a translocation event involving AF6, the human ortholog of Cno, triggers RAS activation (Manara et al., 2014; Smith et al., 2017). Nevertheless, a clear perturbation of Wnt and Notch remains to be proven in this context.

### FGF Signaling

The interaction between Ras/MAPK on the FGF signaling is also normally necessary in various developmental contexts during cell proliferation, migration, and differentiation (Tsang and Dawid, 2004). For instance, in *Drosophila*, multiple interplays were shown that instruct mesoderm migration and muscle specification (Lin et al., 1999). In addition, an intermediary role of FGF signaling in the Ras/MAPK-dependent activity on suppression of Notch-induced HES transcription factors was demonstrated in the context of both insect wings and zebrafish somites’ development (Kawamura et al., 2005). A crucial integration of Ras, FGF, and Notch signaling was also shown for muscle and cardiomyocyte specification (Carmena et al., 2002). Pointing to the importance of a possible dysfunctional interplay between FGF and Ras/MAPK in early embryological events for developmental disease etiology, studies in zebrafish models of CFCS *in vivo* demonstrate that perturbation of gastrulation movements due to hyperactive Ras is rescued by inhibiting FGF signaling (Anastasaki et al., 2009).

On the other hand, a clear crosstalk of FGF on Ras/MAPK also contributes to the balance between cell-type specifications instrumental to brain development. Li et al. (2012) demonstrated that neural stem cells lacking MEK1/2 activity fail to produce glial cells, a mechanism likely acting via modulating an stromal derived factor (SDF-1) input and FGF signaling, as shown for astrocyte development (Bajetto et al., 2001; Song and Ghosh, 2004; Dinh Duong et al., 2019). In addition, it was proven that the regulatory activity of FGF on Ras/MAPK via SOS/Grb2 and Stump seems particularly important for the correct neuromuscular junction (NMJ) formation during *Drosophila* nervous system development via synergistic action of Smn1, which positively regulates FGF pathway components (Sen et al., 2011). In insect models of human spinal muscular atrophy (SMA), a severe autosomal recessive neurodegenerative disease caused by mutation in SMN1 and a primary cause of death in children (Lefebvre et al., 1995), alteration of this interplay seems to contribute to the NMJ defects observed. The impact of a possible dysregulation of Ras/MAPK and FGF for the human SMA condition remains to be assessed.

## RHO Proteins and Their Involvement in Heterogeneous Neurodevelopmental and Hematological Disorders

The RHO family of small GTPases is a large group of proteins (>20) within the RAS superfamily. RAC1, RAC2, RHOA, and CDC42 are classical members regulated by cycling between an active and inactive state via hydrolysis of GTP (Haga and Ridley, 2016; Etienne-Manneville and Hall, 2002; Heasman and Ridley, 2008). Atypical proteins with no intrinsic GTPase activity also exist (for a general survey, refer to Heasman and Ridley, 2008; Ji and Rivero, 2016). By interacting with a myriad of effectors and other small GTPases (Etienne-Manneville and Hall, 2002; Schmidt and Hall, 2002; Ridley et al., 2003; Nouri et al., 2020), RHO proteins control cell polarity establishment and trafficking, cell shape, and motility in health and disease (Ellis and Mellor, 2000; Etienne-Manneville and Hall, 2002; Hall, 2005; Ridley, 2006; Govek et al., 2011, Haga and Ridley, 2016; Lawson and Ridley, 2018; Ueyama, 2019; Boueid et al., 2020), via direct actin-cytoskeletal and MT rearrangements, to generate protrusive and contractile forces by means of filopodia (CDC42), lamellipodia, and stress fibers (RHOA and RAC1) (Etienne-Manneville and Hall, 2002; Heasman and Ridley, 2008; Figure 2). Molecularly, active RHO proteins exert their role by regulating their spatial distribution in the cell (e.g., by shuttling between the cell membrane and the Golgi). Among RHO small GTPases, CDC42 has been extensively studied. By promoting actin-rich filopodia formation via direct activation of N-WASP, Arp2/3, and formin (Etienne-Manneville and Hall, 2002; Mellor, 2010), CDC42 generates polarized cell migration (Govek et al., 2011; Zhang et al., 2014), contributes to various polarized processes underlying morphogenesis, as shown in yeast (Adams et al., 1990; Evangelista et al., 1997; Wedlich-Soldner et al., 2004), nematodes (Kay and Hunter, 2001) and vertebrates (Stanganello et al., 2015). Of note, CDC42 seems to regulate also polar vescicular trafficking as shown in various organisms (Harris and Tepass, 2010).

The importance of RHO proteins in early developmental schemes is illustrated by the embryo lethality often observed in mutant mice models (Sugihara et al., 1998; Liu et al., 2017). As discussed below, particularly important is the impact of RHO proteins on both brain and hematological development. Thanks to the advances of NGS, aberrant Rho signaling caused by mutations affecting multiple genes is emerging also as a prominent cause of clinically heterogeneous neurodevelopmental and hematological rare disorders, which include pediatric cancers. In addition, the numbers of genes (>20) encoding key players of Rho signaling were recently classified as risk factors for autism spectrum disorders by the Simons Foundation Autism Research Initiative, which was backed up by mice models (Guo et al., 2020).

At least 30% of neuroblastoma cases are indeed due to mutations that alter RHO and RAC activity (Dyberg et al., 2017), involved in the migration of neural crest (NC) cells from which neuroblastoma originates. An overview of the genetic conditions linked to RHO proteins is summarized in Figure 2, right (refer to Boueid et al., 2020 for an up-to-date review).

### Brain Formation and Neurodevelopmental Diseases

Extensive work in rodent models highlighted the importance of RHO-dependent neuronal precursors’ mobility and radial glia expansion processes for neuronal circuit establishment and maturation (i.e., corticogenesis) (Govek et al., 2011; Ito et al., 2014; Azzarelli et al., 2015; Xu et al., 2019; Heide et al., 2020). Developmental pathways used by growing axons for initiation, extension, and target innervation depend on Rho signaling *in vivo* (Hall and Lalli, 2010). Accordingly, classical studies using inactive or constitutively active mutants demonstrated the requirements of RAC1-dependent actin remodeling in axon guidance for insect motoneurons innervation (Kaufmann et al., 1998), and similar functions were confirmed in the visual and mushroom body circuits (Hakeda-Suzuki et al., 2002; Ng et al., 2002), as well as in nematode development (Shakir et al., 2006). *Rac1* forebrain knockout (KO) mice showing microcephaly further confirmed a function in vertebrate neuronal migration, proliferation, and dendritic arborization (Chen et al., 2009; Leone et al., 2010).

Of note, the fruitful combination of WES carried on a large cohort of heterogeneous undiagnosed conditions coupled to *in vitro* and *in vivo* functional approaches has recently contributed to map and validate a number of *de novo* mutations in *RAC1* in patients affected by a range of developmental defects, including brain malformations (Reijnders et al., 2017). Similarly, independent studies employing exome and genome sequencing have recently identified also dominant *RAC3* mutations as causative of neurodevelopmental diseases with divergent clinical features, such as the rare Robinow syndrome–like disorder, which shows also impaired skeletal development (White et al., 2018; Costain et al., 2019).

Moreover, experimental evidence shows a fundamental role also of RHOA in cell-type specification of developing brains (Dupraz et al., 2019) and neurite outgrowth (Kouchi et al., 2011; Tan et al., 2020), and this protein was recently involved in a newly discovered syndrome, the “RHOA-related mosaic ectodermal dysplasia,” with clear signs of leukoencephalopathy and anomalies in NC derivatives (Vabres et al., 2019).

Among the other RHO proteins, also CDC42 participates in brain development. The protein regulates polarization and motility in neuronal precursors (Govek et al., 2011; Govek et al., 2018). By acting directly on PAR complex, numb, and E-cadherin, CDC42 orchestrates apicobasal trafficking, spindle orientation, and influences adherens junction integrity, as shown during *Drosophila* neuroepithelium development (Georgiou et al., 2008; Harris and Tepass, 2008) but also in *C. elegans* and other eukaryotes (Gotta et al., 2001; Kay and Hunter, 2001; Harris and Tepass, 2010), and during the normal development of various tissues and organs in mammals (Melendez et al., 2013; Elias et al., 2015). In addition, conditional mice models have been already useful to prove the importance of CDC42 and RAC in the establishment of cell polarity for acquiring the specialized cell morphology in the context of cochlear hair cells (Ueyama et al., 2014; Kirjavainen et al., 2015) and in hippocampal axonal formation (Garvalov et al., 2007).

Lastly, unexplored signaling via MT during migration is seemingly able to activate RHO molecules within a feedback loop, which has a great impact on neuronal polarity establishment (Wojnacki et al., 2014). However, the mechanism awaits confirmatory *in vivo* analysis.

### Hematological Development and Disease

A large body of evidence *in vitro* and in animal model systems demonstrates a crucial function of RHO proteins also in immune cell development and physiology (Nayak et al., 2013).

Rodent models illustrate indeed a unique role of RAC2 for chemoattractant-dependent neutrophil migration and oxygen radical production during immune response to infections (Troeger and Williams, 2013). CDC42 has an equally important role in this developmental context, by controlling the events at the front and back of migratory immune cells via the integration of integrins, WASP protein, CD11b, and MT signaling (Kumar et al., 2012). Confirming the relevance of RHO proteins in hematopoietic cell development and disease, by using WES, several authors have recently established the pathogenicity of various dominantly acting mutations in *RAC2*, which cause pediatric immunodeficiencies affecting T, B, and myeloid cells (including Hsu et al., 2019; Sharapova et al., 2019; Lagresle-Peyrou et al., 2020). These results were also substantiated by mice models (Hsu et al., 2019). Fish models also exist, which showed an involvement of the ortholog version of RAC2 in controlling neutrophils and leukocytes’ behavior (Rosowski et al., 2016). Given the importance of cytoskeletal dynamics in modulating immune development and response, and cell migration in general, it is not surprising that mutations in RHO proteins alter important features of hematopoietic stem cells (HSCs) and contribute to tumorigenic and metastatic processes involving these cells (Maldonado and Dharmawardhane, 2018). *RAC2* genetic lesions were indeed observed in acute myelogenous leukemia (Ross et al., 2004; Thomas et al., 2008). Independent mice models show that active CDC42 causes aging in HSC with impairment in cell polarity and function, a condition that might be linked to aging and myeloid tumorigenesis (Kerber et al., 2009; Geiger and Zheng, 2013). Based on these considerations, inhibiting CDC42 function has been recently proposed as a valid approach to ensure long-term HSC mobilization as a therapeutic tool for various blood diseases (Liu et al., 2019).

### Newly Discovered Syndromes Linked to Aberrant CDC42

Multiple lines of evidence from human disease studies and *in vivo* models are also pointing to a broad impact of dysregulated CDC42 function in various processes, which impact both brain and hematological development. Indeed, Martinelli et al. (2018) linked dominantly acting missense mutations causing variably malfunctioning of CDC42 to an unusually heterogeneous group of developmental conditions (including RASopathy traits), mainly characterized by variable growth dysregulation, neurodevelopmental defects with impaired hearing and vision, and immunological and hematological anomalies. The mutations altering variably the interaction with regulatory and signaling effectors impacted cell migration and nematode vulva morphogenesis, likewise in RASopathies models caused by altered Ras/MAPK signaling.

Noteworthy, a peculiar mutation in *CDC42*, which locks the protein in the Golgi, was more recently proven to be causative of a complex and previously undiagnosed life-threatening autoinflammatory condition, NOCARH syndrome (neonatal onset cytopenia with dyshematopoiesis, autoinflammation, rash, and hemophagocytic lymphohistiocytosis), impairing hematological development (Lam et al., 2019). HSCs of NOCARH patients had reduced responsiveness to proliferation stimuli and immune response due to altered cell polarity (Lam et al., 2019). It would be interesting to test the effect of CDC42 inhibition approach (Liu et al., 2019) on HSC cell behavior of future NOCARH *in vivo* experimental settings.

## RHO-Mediated Developmental Signals Integration in Cell Polarity, Morphogen Distribution, and Relevance for Pathology

Although more investigation is needed, we now know that RHO proteins exert their function also by acting as molecular switches on several signaling pathways during embryogenesis. A schematic overview of some of the main signaling and events influenced by RHO small GTPases is illustrated in Figure 2 (center). Indeed, the clinically broad spectrum of RHO-linked diseases might reflect the pleiotropic impact of RHO proteins’ functions on modulating and interpreting the different signalings. However, the implications for disease etiology are poorly investigated, which calls for systematic functional profiling of the mutations in the context of vertebrate development, currently lacking. We examine here some examples of the available evidence for signaling interplay also in the context of pediatric diseases.

### PCP and Wnt Signaling

RHO requirement for signal integration on non-canonical Wnt signaling–meditated planar cell polarity (PCP) was shown in *Drosophila* mutants defective in wing and eye morphogenesis, as well as in fish, frogs, and other models (Schlessinger et al., 2009). In addition, RHO-dependent actin rearrangement and polarity establishment for PCP-dependent CE cell movements during gastrulation were demonstrated in mammalian cells and in the context of frog and zebrafish gastrulation (Habas et al., 2001, 2003; Marlow et al., 2002). On the other hand, a growing body of data in animal models proves the interplay between RHO and Wnt during embryonic NC migration (reviewed by Mayor and Theveneau, 2013). In this context, Kratzer et al. (2020) have recently provided evidence for a novel and a complex modulatory mechanism acting during frog development, where Rho GEF Trio activates Rac1 at the level of cell protrusions of migratory cranial NC via interaction with Dvl, a major player of PCP signaling (Gao and Chen, 2010), similar to other Dvl-dependent mechanisms seen for Rho activation in *Xenopus*, *Drosophila*, and zebrafish development (Schlessinger et al., 2009). It is also becoming clear that RAC1 is involved in a positive regulation of canonical Wnt signaling by enabling the nuclear accumulation of β-catenin (Wu et al., 2008; Schlessinger et al., 2009).

Similar to what was observed in mammalian cells, classical *Drosophila* wing and eye systems, as well as *Xenopus* embryo models, were useful to demonstrate the necessity of several RHO GTPases in mediating actin cytoskeleton modifications via non-canonical Wnt signaling during development (Strutt et al., 1997; Habas et al., 2003; Mezzacappa et al., 2012).

When it comes to relevance for pathology, the involvement of Wnt pathway alteration in RHO-associated diseases begins now to emerge. For instance, although the molecular mechanism of the *RAC3*-linked Robinow syndrome–like disorder (Costain et al., 2019) remains unsolved, the disease is normally associated with Wnt signaling alterations, which would be interesting to validate *in vivo* functionally in relation to disease etiology (White et al., 2018). In addition, lack of CDC42 results in loss of apical molecules’ distribution throughout rodent telencephalon development, including the canonical Wnt effector β-catenin, and determines Shh-independent holoprosencephaly (Chen et al., 2006).

Lastly, a role for RHOA-dependent kinase alteration underlying non-canonical Wnt (PCP) pathway is emerging also for neuroblastoma (Becker and Wilting, 2019) and B-cell precursor ALL (Karvonen et al., 2019). Recent work on KRAS-G12D–induced zebrafish models of embryonal RMS and *in vitro* human RMS has demonstrated a crucial role of hyperactive RHOA in the promotion of tumor propagating cell self-renewal downstream Vangl2, a classical non-canonical Wnt regulator, under a similar molecular axis known in embryogenesis (Hayes et al., 2018).

### FGF and VEGF Signaling

RHO proteins are able to integrate a number of other signalings relevant in different contexts during normal embryonic development. Their activity is essential for orchestrating the molecular dynamics needed for cell shape changes, for example, by assisting actin–myosin ring formation for apical cell constriction during gastrulation (Nikolaidou and Barrett, 2004; Dawes-Hoang et al., 2005). Experiments in *Drosophila* models showed that the modulatory activity on FGF signaling contributes substantially to these processes and ultimately to mesodermal cell motility (Smallhorn et al., 2004). Specifically, it was demonstrated that insect Rho GEF pebble, normally involved in Rac1 and Rac2 activation (van Impel et al., 2009), can modulate FGF-mediated Ras/MAPK signaling to establish EMT conversion and mesodermal cells migration (Smallhorn et al., 2004).

On the other hand, RHOA is fundamental for the reorganization of the F-actin during cytoskeleton remodeling in endothelial cells and angiogenesis, which is highly relevant for pathology onset and progression (Merajver and Usmani, 2005). Importantly, both *in vitro* and *in vivo* models showed that cytoskeletal dynamics tuned by RHO molecules act under direct VEGF signaling to impact migratory movements and trafficking of endothelial cells during angiogenesis (Soga et al., 2001; van Nieuw Amerongen et al., 2003; Garret et al., 2007; Lamalice et al., 2007; El Baba et al., 2020). In this regard, inhibitors acting on the RHO/ROCK-mediated pathway are becoming a promising therapeutic approach for vascular endothelial growth factor (VEGF)-induced angiogenesis in the context of tumor progression and invasion (van der Meel et al., 2011; Chen et al., 2014).

### PI3K Signaling

Experiments with photoactivable RHO versions in zebrafish demonstrated a direct involvement of Rho proteins in cell polarity, actin dynamic, and migration in neutrophils by the activity of PI3K signaling (Yoo et al., 2010). While demonstrating a crucial involvement of SDF-1/CXCR4-mediated Rac2 activity in limiting neutrophil mobilization, zebrafish models of primary immune deficiency caused by human *RAC2* mutations or morpholino approaches indicate that the pathogenic role for *RAC2* in immune cell physiology (Hsu et al., 2019; Sharapova et al., 2019; Lagresle-Peyrou et al., 2020) is linked to an altered PI3K-mediated cell polarity signaling in neutrophil migration during an inflammatory response (Deng et al., 2011). On the other hand, confirming the relevance of RHO modulatory activity on developmental signaling also for cancer, mutations in *RHOA* have been recently linked to B-cell lymphoma and Burkitt lymphoma via impaired PI3K pathway (Svensmark and Brakebusch, 2019; Voena and Chiarle, 2019).

Even a complex interplay between Ras and Rho signaling involving PI3K/AKT pathway and likely also canonical Wnt via GSK3-β modulation was shown in rat hippocampus neurons. A fine balance of signaling output in this crosstalk ensures the activity of CDC42 and RAC for regulating MT dynamics during axon initiation (Schwamborn and Püschel, 2004; Hall and Lalli, 2010).

The modulation of PI3K seems relevant also for brain tumor onset, as demonstrated by the increased expression of CDC42 through the PI3K/AKT/N-myc signaling pathway, which correlates with undifferentiated childhood neuroblastoma (Lee et al., 2014).

### CDC42-Controlled Morphogen Distribution

Of particular interest for development and disease are *in vivo* findings demonstrating a unique mechanism by which CDC42 acts as a special signaling node for pathways directly regulating morphogen distribution in different developmental contexts. *In vivo* fish studies have demonstrated that Cdc42/N-Wasp filopodia act as “signaling extensions,” allowing fine control of morphogen propagation during development. Elegant genetic and imaging experiments in zebrafish embryos show that Cdc42-dependent filopodia determine short- and long-range propagation of canonical Wnt signaling and paracrine signal activation during vertebrate gastrulation, with major impact directly on the anterior–posterior (AP) axis and neurogenesis (Stanganello et al., 2015). This unique function of CDC42-induced filopodia was shown to contribute also to Hh signaling during avian tissue patterning (Sanders et al., 2013). In addition, work with zebrafish transgenic tools labeling intracellular structures and reporting and modulating Cdc42 activity carried at the single-cell precursor level *in vivo* suggested a BMP control of Cdc42-enriched filopodia necessary for *in vivo* endothelial cell motility during angiogenic sprouting (Wakayama et al., 2015). It is worth mentioning that control of morphogen asymmetric distribution, polarity, and signaling modulation seem to depend on RHO-like proteins also during plants’ root hair formation, suggesting a deeply conserved function in organismal development (Wu et al., 2011).

## ARF and RAB-Mediated Biosynthetic Trafficking and Involvement in Pediatric Diseases

Biosynthetic trafficking is a highly conserved process crucial for setting signaling coordinates during organismal development and physiology (Biechele et al., 2011; Fernandez-Valdivia et al., 2011; Shimokawa et al., 2011). A number of proteins regulate and participate in this process, among which the small ARFs, including several classes, ARF-like (ARL) and RAB GTPases (>70 proteins in humans), which we discuss here, as well as related SAR proteins (Kahn et al., 2006). Mechanistically, like the other small GTPases, the function of ARF and RAB small GTPases is controlled via cycling between the GTP and GDP-bound forms by the action of specific GEFs and GAPs (Barr and Lambright, 2010; Sztul et al., 2019). Many of these proteins show a GTP hydrolysis-dependent spatial shuffling between cytoplasm and Golgi apparatus (GA), which is crucial to regulate important steps in the endoplasmic reticulum (ER)–GA network together with RAB proteins, such as coat proteins recruitment, vesicles biogenesis, cargo sorting, and signaling (Palmer et al., 1993; Reinhard et al., 2003; Figure 3). For a comprehensive review of ARF and RAB biochemistry, function, and on the role of membrane dynamics in development, refer to Wandinger-Ness and Zerial, 2014; Wada et al., 2016; Sztul et al., 2019; Marwaha et al., 2019.

Several ARF proteins, divided in three major classes based on their sequence homology, are involved in various steps of the intracellular trafficking, recruiting effectors for vesicle formation, budding, tethering, and cargo sorting as modulating actin and MT-based cytoskeleton (Sztul et al., 2019). On the other hand, RAB proteins distribute to specific subcellular compartments in combination with other proteins (e.g., tethering complex proteins or SNARE) to control vesicles formation and fusion via interaction with cytoskeleton components essential in development (Hutagalung and Novick, 2011; Wandinger-Ness and Zerial, 2014). These small GTPases have broad functions during development, ranging from gastrulation events to differentiation. Manifesting the importance of ARF and RAB during these processes, mutations affecting the function of these proteins, their regulators, and effectors have a deleterious effect on development and contribute to human pathology, with an important impact on the nervous system formation, as discussed below. In Figure 3, right, an overview is shown of the main genes (encoding key proteins belonging to or interacting with members of the ARF and RAB family) whose mutations have been associated with developmental diseases in which organelle’s dynamic and morphogen distributions are altered.

Further highlighting the extended domain of action in development and physiology, mutant RAB proteins also cause inherited pediatric immunodeficiencies (Griscelli and Prunieras, 1978) and in cancer. As an example, alterations of *RAB15* alternative splicing, for instance, were linked to neuroblastoma tumor-initiating cells (Nishimura et al., 2011; Pham et al., 2012). In cancer, the aberrant activity of these proteins can modulate negatively various tumorigenic steps (including metastasis), where they can work both as oncogenes and tumor suppressors (a role recently reviewed by Casalou et al., 2020 and Gopal Krishnan et al., 2020). Besides the cases discussed below, the role of ARF and ARF-related proteins in animal development was recently reviewed in pathological contexts by Rodrigues and Harris (2019).

### Maintenance of GA Integrity and Cytoskeleton Physiology by ARF

Of particular interest is the role of ARF proteins in the maintenance of organelle integrity, cytoskeleton remodeling and dynamics (Myers and Casanova, 2008; Kondo et al., 2012), highly relevant processes for organism development, brain formation, and neuronal circuits’ function, and which are involved in the onset of neurodevelopmental pathologies. Indeed, hyperactive *ARF1* mutations induce loss of GA structure and fragmentation, which is likely mediated by COPI + vesicle budding in both healthy and diseased tissues (Zhang et al., 1994; Xiang et al., 2007). In fact, a series of genetic diseases affecting the developing nervous system caused by ARF and GA structural and functional alterations are emerging as “golgipathies” (Dupuis et al., 2015; Rasika et al., 2018) whose mechanisms need to be explored. Cancer-related GA fragmentation, often coupled to an increase of Ras/MAPK signaling and likely to alteration of ARF function, might even be a promising therapeutic target (Petrosyan, 2015).

Interestingly, GA alterations are also a hallmark of common forms of adult neurodegenerative processes (Rabouille and Haase, 2016), and cancers (Petrosyan, 2015) and ARF-mediated ER–GA trafficking perturbation were demonstrated in amyotrophic lateral sclerosis (ALS) (Zhai et al., 2015; Atkin et al., 2017). Of note, in nematode, superoxide dismutase 1–ALS disease models Arf proteins might even have a protective function on neurons (Zhai et al., 2015).

Moreover, employing rodent disease models, Bellouze et al. (2014) proved clear crosstalk between ARF-dependent trafficking and MT, which causes GA disintegration and is likely responsible for early onset neurodegeneration with progressive motor neuropathy (Schaefer et al., 2007; Sferra et al., 2016). Despite that further investigation is needed, it is tempting to speculate that a lack of function in the tubulin cofactor proteins (i.e., TBCE) in the affected children (with a possible loss of interaction with ARF1) (Bellouze et al., 2014) contributes to motoneuron degeneration (Schaefer et al., 2007; Sferra et al., 2016).

Via a specific GAP protein (RP2), also ARL2 and ARL3 influence MT dynamics and GA stability, as well as protein trafficking to the cilium, with a major impact on photoreceptor development in mice (Evans et al., 2010; Schrick et al., 2006). Accordingly, mutations affecting the ARL3 GAP protein RP were long shown to cause a severe form of X-linked retinitis pigmentosa, linked to altered GA stability and impaired trafficking to the photoreceptors’ cilia (Schwahn et al., 1998). Consistently, retinal degeneration was observed in *Arl3* KO mice (Schrick et al., 2006), and mutant *arl3* alters ciliogenesis in *C. elegans* (Li et al., 2010), with an important consequence on cilia signaling as discussed below.

Lastly, evidence for a crosstalk between ARF1 and actin–cytoskeleton regulators of RHO family was also shown. ARF1-mediated assembly of COPI complex is crucial for recruitment of CDC42 to the GA and for the local activation of N-WASP, Arp2/3, and actin polymerization, necessary to promote vesicle formation and scission (Wu et al., 2000; Myers and Casanova, 2008). An ARF1–RAC interaction was also shown for recruiting to the membrane WAVE (WASP family) and actin polymerization (Koronakis et al., 2011).

## ARF and RAB Proteins’ Contributions to Set Signaling Coordinates Across Developmental Fields and Relevance for Pathology

Acting on shaping developmental signals strength and distribution and regulating cell behavior, ARF and RAB-controlled biosynthetic trafficking globally modulates morphogens’ distribution and function in developmental programs. The regulated process of polarized vesicular transport of morphogenes guarantees coordinated cell–cell signals and movements during development, ultimately leading to cell specification and organogenesis (Eaton and Martin-Belmonte, 2014; Wada et al., 2016; Rodrigues and Harris, 2019). Indeed, an aberrant function of ER and GA enzymes impairs proteins’ modification, with a deleterious impact on Wnt, Notch, and FGF signaling, as shown in insects and rodents (Biechele et al., 2011; Fernandez-Valdivia et al., 2011; Shimokawa et al., 2011). Furthermore, endosomes are emerging as important signaling platforms, mediating canonical Wnt signaling (Blitzer and Nusse, 2006) and sorting TGF-β signaling outcomes (Di Guglielmo et al., 2003; Felici et al., 2003). Examples of the direct influence of ARF and RAB function on developmental signaling in organismal context is also available (some of the main examples are schematized in Figure 3, center).

Here, we examine established and mounting evidence on the direct action of ARF and RAB-mediated intracellular trafficking on crucial developmental pathways, relevant for morphogenesis and disease. In general, these proteins have an active role in intracellular trafficking and cytoskeleton reorganization in cilia formation, directly regulating cilia development (Fisher et al., 2020), their general function, and cilium-dependent signaling pathways, including Shh and Wnt during organogenesis, as proven in different model systems (Huangfu and Anderson, 2005; Eggenschwiler et al., 2006).

### PCP and Wnt Signaling

Despite poor mechanistic understanding, a clear contribution of ARF function in the modulation of both canonical and non-canonical Wnt signaling is emerging to be directly relevant for pathology.

Many developmental processes including proliferation and differentiation require controlled ARF-dependent biosynthetic pathways for establishing cell polarity. This was demonstrated in several developmental models and timings, i.e., gastrulation events (Lee et al., 2015), dendritic spine formation and growth in vertebrate hippocampal neurons (Jain et al., 2012), insect neuronal maturation (Chang et al., 2015), photoreceptor differentiation (Mazelova et al., 2009; Wang et al., 2012), and bone formation (Unlu et al., 2014), and is confirmed by genomic studies of developmental conditions.

Activating mutations affecting *ARF1* result in a complex neurodevelopmental condition called “periventricular nodular heterotopia.” This neuronal migration disorder is characterized by microcephaly with brain malformations and progressive cerebral atrophy and spasticity (Ge et al., 2016), and invertebrate and vertebrate embryo models expressing dominant *ARF1* exist, which show typical non-canonical Wnt-dependent PCP defects (Carvajal-Gonzalez et al., 2015). Specifically, solid experiments in the *Drosophila* wing model showed that Arf1, together with the Ap-1 adaptor complex, is instrumental for setting PCP during cell specification. Via direct control of Frizzled trafficking, Arf1 is majorly responsible for the restricted polarized accumulation of the signaling complexes formed by frizzled/disheveled/Diego and Van Gogh/prickle (Vang/Pk) within a single precursor cell, which guarantees correct morphogenesis. Furthermore, although the exact mechanism remains unproven in vertebrates, suggestive of the decisive impact on complex vertebrate embryogenesis events, constitutively active Arf1 (obtained by overexpressing the human variants) results in typical PCP-dependent phenotypes in zebrafish, i.e., body shortening and morphological alterations of the AP axis, likely caused by perturbed gastrulation cell movements (Carvajal-Gonzalez et al., 2015). Furthermore, work in *C. elegans* suggests a novel mechanism for both ARF and RAB small GTPases involving the modulation of a special non-canonical Wnt signaling that uses β-catenin for asymmetric divisions during development (Hardin and King, 2008).

On the other hand, canonical Wnt signaling might also be mediated by ARF-trafficking activity during development. Active ARF1 and ARF6 stimulate the production of PtdIns (4,5) P2 (Godi et al., 1999; Honda et al., 1999), which activates the Wnt coreceptor LRP6 (Zeng et al., 2005), resulting in hyperactivation of canonical Wnt signaling (Zhang et al., 2007). Consistently, it was shown that the function of specific ARF GEFs (such as BIG2) is essential for β-catenin distribution and activation in human cortical development (Sheen et al., 2004). *Vice versa*, even a positive control of canonical Wnt on ARF was demonstrated (Kim et al., 2013). An involvement of Wnt signaling modulation might underlie the X-linked mental retardation (Shoubridge et al., 2010) caused by missense mutations in *IQSEC2* (encoding an ARFGEF specific for ARF6) and the complex and pleiotropic ciliopathy Bardet–Biedl syndrome showing polarity defects, associated with ARL6 (Wiens et al., 2010). Mutations affecting directly the ARFGEF BIG2 protein, fundamental for β-catenin action during brain development, were observed in children with autosomal recessive periventricular heterotopia manifesting severe cerebral cortex malformations and microcephaly, also likely underlying impaired Wnt signaling and impaired neuronal cell migration (Sheen et al., 2004).

Evidence exists also for a modulation of various aspects of Wnt signaling by members of the RAB family. It was shown that, by regulating the internalization of LRP6 receptor, RAB8B can control Wnt signaling. Confirming the *in vitro* data, a lack of Rab8b was found to block Wnt signaling during fish development (Demir et al., 2013). Moreover, RAB23 was implicated in positively regulating Wnt11/AP-1 signaling in a mechanism mediating C-Jun N-terminal kinase, contributing to cardiomyocyte differentiation in fish models (Jenkins et al., 2012).

By controlling the generation of endocytic compartments, precursor cells can regulate their fate in embryonic developmental fields to shape tissue formation. Demonstrating further the importance of RAB-cargo transport in embryogenesis and signaling, Winter et al. (2012) showed a role of Rab11-enriched recycling endosomes for regulating epithelial Par5-dependent polarity in nematodes, whereas Ulrich et al. (2005) clarified a new mechanism for non-canonical Wnt11 activity during zebrafish gastrulation, which functioned via E-cadherin–mediated cell cohesion and establishment of PCP through Rab5-dependent recycling. In the context of pathology, among the genes recently associated with Hirschsprung disease (HSCR), showing impaired enteric nervous system development (Gui et al., 2017), WES analysis identified mutations affecting the GEF DENND3, typically involved in intracellular trafficking by activation of RAB12. Functional investigation using zebrafish morpholino and CRISPR/Cas approach already exists, which supported the function of the fish ortholog in enteric nervous system development (Gui et al., 2017). It would be interesting in this context to also test the functional link between perturbation of RAB activity and Wnt signaling during NC migration for the onset of the pathology, as suggested by zebrafish *ovo1* mutants (Piloto and Schilling, 2010).

### FGF, EGF, and VEGF Signaling

During nervous system development, Schwann cells have a crucial role in responding to a number of signaling and reshape their morphology to form myelin. Mice models show that a specific ARF1 and ARF6 GEF (cytohesin) is involved in this morphogenetic process (Yamauchi et al., 2012), as well as RAB proteins (Stendel et al., 2010). Mechanistically, conditional KO mice provided evidence for ARF6-controlled FGF signaling, which impacted central nervous system (CNS) morphogenesis and myelin formation itself. Indeed, specific lack of ARF6 in rodent neurons resulted in a reduced size of the corpus callosum and of the hippocampal fimbria, underlying impaired secretion of the guidance factor FGF2. This results in defective oligodendrocytes migration and thereby axonal myelination (Akiyama and Kanaho, 2015). Moreover, experiments in mice models of KIF16B loss of function, which recapitulate FGFR2 KO animals, demonstrate that a KINESIN/RAB14 complex mediates Golgi-to-endosome trafficking of the FGFR and that this is crucial for epiblast development (Ueno et al., 2011). Evidence for an involvement in regulating also EGF- and VEGF-mediated signaling events during development emerges from animal models. Indeed, beyond its participation in insect insulin signaling pathway (Fuss et al., 2006), the *Drosophila* ortholog of the ARF-GEF cytohesin was shown to modulate EGF-mediated Ras/MAPK signaling in the context of wing growth and vein morphogenesis, as well as in and eye formation (Hahn et al., 2013). On the other hand, a recent study employing *in vitro* systems and zebrafish has proven the importance of Big2 in angiogenesis, likely depending on the Arf1-controlled VEGF signaling (Lu et al., 2019). The exact mechanism underlying these signalings’ interplays and the relevance for pathology remain to be addressed.

### Shh Signaling

Alteration of cilium-related structure, function by perturbed ARF and RAB-related activity can impact on a number of other signaling pathways influencing brain formation and likely involved in neurodevelopmental diseases. As an example, we know that mutations in *ARL13b* are lined to altered Shh signaling and underlie Joubert syndrome, a condition causing midbrain–hindbrain developmental abnormalities and various other defects typical of impaired Shh signaling (Doherty, 2009). Importantly, mouse and zebrafish models for this condition are available, which can be employed to further investigate the mechanism (Larkins et al., 2011; Zhu et al., 2020). Null mice models for *arl3* have a defective cilium-dependent signaling influencing different pathways, including Shh (Horner and Caspary, 2011) and show retinal degeneration (Schrick et al., 2006). A large amount of data strongly support the involvement of many RAB proteins and effectors in pathogenic alteration of cilium-mediated signaling, which is worth to investigate further (for a comprehensive review on the topic, refer to Oro, 2007; Yoshimura et al., 2007; Banworth and Li, 2018). In this context, the Carpenter syndrome, which harbors prominent neurological features and craniofacial and cardiac malformation, is an example. This condition is caused by mutations in *RAB*23, which is also known to regulate Shh signaling via controlled trafficking to the primary signaling center of the cilium (Boehlke et al., 2010). Accordingly, altered RAB23 in mouse models shows Shh-dependent ventralization defects and altered patterning of neural cell types during spinal cord development (Eggenschwiler et al., 2001; Eggenschwiler et al., 2006). It remains to be proven whether an impaired Shh signaling is involved also in the etiology of the Bardet–Biedl syndrome via the activity of Rabin 8 (a specific RAB8 GEF) (Oro, 2007). Of note, given that alteration of Shh signaling is common in various serious cancer conditions also in the adult, it would be interesting to test the potential of blocking the signaling acting directly on RAB proteins activity.

### Notch Signaling

Lastly, it is well known that RAB-dependent endocytosis contributes to the regulation of the number of Notch/Delta molecules present on precursor cells’ surface and thereby of Notch directional signaling, which is normally fundamental for cell commitment and cell identity, as discussed above. Various mechanisms have been implicated in the regulation of Notch signaling during development. To mention few examples, Notch receptor activation is mediated by RAB5-positive early endosomes in dividing sensory organ precursors of *Drosophila* during asymmetric cell division, which instructs cell specification, tissue growth, and morphogenesis (Coumailleau et al., 2009). Also Rab11-dependent recycling of the specific Notch effector Delta is involved in this process in insects and mammalian cells (Emery et al., 2005) and in general, both Rab1 and Rab11 seem to regulate Notch signaling in *Drosophila* (Charng et al., 2014). Insect mutant screening based on wing morphogenesis identified also RAB7 and RAB8 orthologs as major positive modulators of Notch signaling activity (Court et al., 2017).

## Established and Emerging Tools for *in vivo* Interrogation of Small GTPases Shaping Developmental Dynamics in the Vertebrate Zebrafish Model

To progress our knowledge on rare diseases and boost precision therapy, appropriate *in vivo* tools are required to assess the impact of the identified genetic lesions and map the spatiotemporal alterations of developmental pathways at an organismal level. Animal models, now equipped with unprecedented genomic and imaging-based possibilities, are irreplaceable. When zebrafish or *C. elegans* are used, the workflow is even time- and cost-efficient. As shown, animal models allow us to validate the impact of the identified genetic lesion on a global pathophysiological level, inferring readily altered pathways via established developmental paradigms (e.g., the fly wing system or the CE movements of vertebrate gastrulation), while discovering novel mechanisms on multiple developmental contexts and tissues simultaneously. The utility of animal models also relies on the possibility of setting up preclinical systems for assessing potential targeted treatments, identifying development and physiology principles that can uncover evolutionary rules. Moreover, sophisticated xenografting *in vivo* models enable innovative studies of cancer cells’ heterogeneity (Kim et al., 2017) and investigation of pediatric tumors (Rokita et al., 2019).

Besides the aforementioned assets, different biosensors, fluorescent-based reporters, and actuators superior to classical biochemical approaches are becoming available to use *in vivo* for a highly resolved real-time investigation of small GTPases dynamics. These tools allow the visualization and manipulation of small GTPases’ activity in a controllable manner, directly in the developing tissues of entire organisms, thus expanding the possibilities to answer mechanistic questions. Concurrent advances in the development of fluorescent proteins are rapidly accumulating toward the development of near-infrared (NIR) emitting molecules that improve the light penetration in deep tissues with little scattering (Shcherbakova et al., 2018b). Furthermore, advanced modalities for deep tissue imaging are also exponentially becoming available (i.e., two- and three-photon microscopy and a range of optoacoustic modalities) (Helmchen and Denk, 2005; Deán-Ben et al., 2016; Shcherbakova et al., 2018b). Nevertheless, the application and visualization of reporters’ dynamics and the use of genetically encoded actuators in large species remain challenging.

Because it embodies all these advances *in vivo*, zebrafish is once more forcefully becoming a convenient system for rare disease research. Numerous illustrative examples for zebrafish models of diseases underlying a functional dysregulation of small GTPases exist. Among those, notorious RASopathy models are available (Jindal et al., 2015) and RHO-associated developmental syndromes (Boueid et al., 2020). Typical advantages of zebrafish include the high fecundity, rapid development, and a rich community distributing transgenic lines and forward and reverse genetics mutants of various players involved in developmental signalings. Sophisticated genetics and a range of synthetic biology applications as well as imaging innovations discussed below, which allow monitoring of fast subcellular events at nanoresolution, are being quickly implemented in this model. Altogether, these tools are uniquely valuable to dissect real-time altered signaling dynamics throughout embryogenesis with single-cell precision, which is directly translatable to humans.

### Genetic and in *in vivo* Imaging Advances

Zebrafish is especially amenable to gene perturbation for both loss- or gain-of-function genetic alterations via transient approaches (morpholino-based gene knockdown and gene overexpression) or strategies for stable modifications (TALENs and CRISPR-Cas9-based genetic engineering, Hwang et al., 2013). This allows the generation of models for the genetic diseases with a fast phenotyping at different levels, which can be obtained even in 2 days from the microinjection in F0 animals (Wu et al., 2018). Noteworthy, thanks to the continuous optimization of the Base Editor–CRISPR/Cas technology, it is now becoming possible in zebrafish to refine diseases’ modeling even toward patient-specific endeavors, obtaining inheritable precise single-nucleotide conversions (Qin et al., 2018; Rosello et al., 2021).

As far as *in vivo* functional imaging is concerned, zebrafish embryos show far fewer constraints as compared to rodents. They develop externally and are mostly transparent such that cellular dynamics can be readily resolved under fast microscopes in the whole-organism (Wolf et al., 2015; Abu-Siniyeh and Al-Zyoud, 2020), allowing, for instance, accurate brain-wide mapping of calcium fluxes (Renninger and Orger, 2013), even at the level of the whole adult brain (Deán-Ben et al., 2016; Chow et al., 2020). Moreover, a repertoire of behavioral readouts is available that can be implemented to evaluate intellectual delays and complex cognitive deficit, modeling, for instance, RASopathies traits (Wolman et al., 2014). Cancer models based on live imaging of xeno-transplanted malignant cells are also being successfully employed (Cayuela et al., 2019), whereas optimization of the Nobel-worth super-resolution structured illumination microscopy is now being experimented to image live brains in zebrafish (Turcotte et al., 2019), and more advanced imaging possibilities are currently unrolling.

Combining genetics and imaging advances, it is possible to follow molecular and cellular dynamics of virtually any developing tissue. mRNAs encoding fluorescent markers, which emit in a wide range of wavelengths and label-specific cell compartment, can be readily coinjected at early embryonic stages and offer the possibility for multiplexing live imaging, from the very early blastula and gastrula stages, to create mosaic expression both for overexpression and cell-labeling studies. Testifying the efficacy of these simple tools for investigation of *in vivo* developmental signaling, coinjection of mRNA encoding the membrane marker mCherry-GPI together with Wnt8-eGFP permitted to infer canonical Wnt transport and its paracrine activity mediated by Cdc42/N-Wasp + filipodia during early zebrafish development (Stanganello et al., 2015). Moreover, a variety of transgenic and enhancer trap lines are available, and effective fluorescent reporters for major signaling pathways are routinely utilized in zebrafish, including Wnt (Facchinello et al., 2016) and Hh (Mich et al., 2014). Semitransparent pigment mutants used as background in imaging applications (Antinucci and Hindges, 2016) are useful for dissecting the impact of disease-causing mutations on specific anatomical districts (Tabor et al., 2019) even in juvenile and adult fish.

### Cell Lineage Tracing Tools and Signal Perturbation With Photosensitive Proteins

Special transgenic fish exist for specific and dynamic cell lineage tracing, which employ a large variety of photosensitive proteins controlled by light (Chow and Vermot, 2017). Among these, photoconvertible fluorescent proteins such as KikGR (Lombardo et al., 2012) and Kaede are employed to trace various neurons, axons, and circuits (Sato et al., 2006) or migratory cells in the context of retina development and morphogenesis (Kwan et al., 2012). Together with sophisticated Cre-based multicolor (*multibow*) barcoding strategies (Xiong et al., 2015), these tools expand the available palette and allow following movements and ontogeny of a specific subset of cells and their derivatives throughout development in fish models of genetic diseases. Thereby, the origin of cellular organization in rather complex organs and tissues and their alterations can be tackled *in vivo.*

Proof of the possibility to capture even the signaling pathway history in a subset of zebrafish cells during development using photoconvertible proteins exists. The PHotoconvertible REporter of Signaling History (PHRESH) method using Kaede under the control of *ptch1* regulatory sequence allowed indeed mapping temporal dynamics of Hh signaling during cell fate decision in fish spinal cord (Huang et al., 2012). Moreover, sophisticated photochemistry approaches using synthetic and genetically encoded photoactivable probes are being vastly implemented to study several developmental biology mechanisms in animal models, including zebrafish (Kowalik and Chen, 2017).

### *In vivo* Reporters, Actuators, and Transgenic Lines to Study Small GTPASES Activity

Reporters and actuators to monitor and manipulate small GTPases are being developed for fish, which can be combined with the aforementioned tools. In this context, different strategies have been established, with remarkable applications *in vivo* (schematized in Table 1).

**TABLE 1:** Advanced genetic reporters, markers, and actuators to study RAS-, RHO-, and ARF/RAB-dependent processes in the context of live cells and animal development.

Reporters, markers, and actuators	Target	Description
FRET-based EKAR-type biosensors *Tg[ef1*α*:ERK biosensor-nes] (Teen)* transgenic zebrafish line	ERK	Fluorescence resonance energy transfer (FRET)-based ERK biosensors employed *in vitro* and *in vivo* (Fritz et al., 2013) *Used for mapping spatiotemporally Erk activity during embryonic development in zebrafish blastula, gastrula and segmentation stage (Wong et al., 2018)*

ERK and PKA FLIM sensors (EKARet)	ERK PKA	Highly sensitive sensors for monitoring ERK and PKA activity by optimizing the FRET pair for two-photon fluorescence lifetime imaging microscopy (2pFLIM). Useful in the context of rodent structural neuronal plasticity (i.e., long-term potential) (Tang and Yasuda, 2017)

FLIM-based RAS sensor	RAS	FLIM-based RAS biosensor for two-photon imaging used to study RAS signaling during long-term potentiation in hippocampal neurons (Yasuda et al., 2006)

KTR-based ERK sensors (i.e., ERK-nKTR)	ERK	Kinase translocation reporters for measuring nuclear ERK activity *in vitro* and *in vivo*. Accurate reporting MPK1 activity (ERK ortholog in *C. elegans*) in different cell types/developmental processes *in vivo* such as polarized epithelial cells, migrating muscle cell precursors, sensory neurons, and germ line development (Regot et al., 2014; Maryu et al., 2016)
*Tg (ubi:ERK-KTR-Clover)^*vi28*^ (DREKA)* transgenic zebrafish line		*Useful for determining Erk dynamics during zebrafish muscle wounding and establishing small compounds kinetics (Mayr et al., 2018)*

RAF-FLUC bioluminescent biosensor	RAF1 reporter downstream Ras/MAPK signaling	Monomolecular bioluminescent biosensor based on split *firefly* luciferase complementation approach for imaging endogenous RAS activity *in vitro* and *in vivo*. Employed in xenograft mouse models to determine the effect of mutant Ras activity and the responsiveness to treatments (Chen et al., 2017)

*Photoswitchable MEK (psMEK)*	*MEK1 actuator downstream Ras/MAPK signaling*	*Dronpa-based photoswitchable mitogen-activated protein kinase 1 enzyme (psMEK) for exploring Ras/MAPK cascade in vivo. Employed in Drosophila and zebrafish to manipulate Ras/MAPK signaling during gastrulation and expandable to test the strength in signaling activation of gain of function MEK disease-associated mutations (Patel et al., 2019)*

OptoSOS	ERK	Optogenetic actuator to modulate Ras/MAPK activity using light-inducible dimers (iLD). Utilized in *Drosophila* to study the tolerance of early stem cells to increased ERK signaling during gastrulation and cell fate decision (Johnson et al., 2017; Johnson and Toettcher, 2019)

FRET-based RHO biosensors	RHO	FRET approach useful to measure the activity of several RHO GTPases including CDC42, RHOA and RAC. Used in *Xenopus* embryos to evaluate neural crest cells directional migration (Matthews et al., 2008)

FRET sensor Raichu-RHO combined with the photoactivable RHO version (PA-RHO)	RHO	Advanced FRET-biosensor imaging technology to study the activation dynamics of RHOA, CDC42, and RAC1. The combination of PA-Rac1 and Raichu-Rac1 biosensors was useful in the context of cell migration in *Xenopus* and *Drosophila* embryos (Wang et al., 2010)
*PA-Rac1*	RAC1	*Optogenetic tool to induce Rac1 activation was used to investigate neutrophils-mediated immune response in zebrafish (Yoo et al., 2010). Specific transgenic line generated to express PA-Rac1 in developing motoneurons used in zebrafish to control axonal growth and correct axonal guidance defects in plod3^–/–^ mutant fish (Harris et al., 2020) Improved deconvolution algorithm with stepwise optical saturation microscopy (DeSOS) approach used in zebrafish for studying Rac1 function in mediating actin remodeling and filopodia stabilization during sensory neurons axogenesis (Zhang et al., 2019)*

Near-infrared (NIR)–FRET biosensor	RAC1	Smart combination of RAC1 with cyan fluorescent protein–yellow fluorescent protein (YFP) FRET biosensors for RHOA and RAC1–GDP dissociation inhibitor (GDI) binding. Used concomitantly with LOV-TRAP optogenetics is advantageous for observing and quantifying antagonist actions of RHOA and RAC1 on the RHOA-downstream effector ROCK in the context of cell motility (Shcherbakova et al., 2018a)

New-generation intensiometric small GTPase biosensors	All small GTPases	Red-shifted sensors combined with blue light–controllable optogenetic modules allow visualization and manipulation of GTPases activity in a highly spatiotemporal manner in single cells *in vivo* (e.g., structural plasticity of neuronal dendritic spines). Used to monitor subcellular Ras activities in the brains of freely behaving mice (Kim et al., 2019)

GAL/UAS-based transgenic RHO reporter fish Cdc42: *10xuas:mCherry-F2A-myc-Cdc4*2 Rac1: *10xuas:EGFP-F2A-Rac1* RhoA: *10xuas:mCherry-F2A-myc-RhoA*	Several RHO	*Stable lines employed for in vivo visualization of wild-type and mutant Rho signaling dynamics in zebrafish (Hanovice et al., 2016)*

Cell-specific transgenic fish *Mpx/mpeg:mCherry-2A-rac2 Mpx: mCherry-2A-rac1*	RAC1 RAC2	*Stable lines with specific expression in zebrafish macrophages and neutrophils (Deng et al., 2011; Rosowski et al., 2016) used to study Rac-mediated cell motility in host defense mechanisms*

LOV-domain–based inducible RAC1		Optogenetic actuator to control RAC1 activity *in vivo* (Wu et al., 2009). The tool was useful to investigate the function of RAC1 in cell polarity and migration of *Drosophila* ovary (Wang et al., 2010) and to study dynamics of neutrophils activity in zebrafish (Yoo et al., 2010)

*UAS:Myr-GFP-ACK42*		*Stale line expressing a specific inhibitor used in vivo to investigate the contribution of Cdc42 to endothelial cell motility during angiogenesis in zebrafish (Wakayama et al., 2015)*

Biomolecular Luminescence Complementation (BiLC) biosensors	RHO	Bioluminescent sensors, based on genetically engineered *firefly* luciferase, enable non-invasive visualization and quantification of RHO activity in mouse (such as in tumor models) and for *in vivo* imaging and high-throughput screening of therapeutic drugs targeted to RHO (Leng et al., 2013)

FRET-sensor Raichu-Rab5	RAB5	FRET-sensor based on Venus/SECFP FRET pair used to study Rab5 activity during phagocytosis in mammalian cells (Kitano et al., 2008)

COnformational Sensors for GTPase Activity (COSGA)	RAB1 RAS	Universally applicable conformational FLIM-based sensors for monitoring RAB1 and KRAS activity in live cells. Used for quantitative analysis of small GTPases activity at high spatial and temporal resolution (Voss et al., 2016)

IM-LARIAT	Several RAB	Optogenetically controlled RAB actuator based on the blue light–sensitive cryptochrome 2 (CRY2) used in rodent hippocampal neurons to study various aspects of intracellular trafficking depending on specific RAB in dendritic maturation (Nguyen et al., 2016)

Transgenic toolkit	Small GTPases-controlled intracellular processes	
*Rab5*, *Rab7*, *Rab11*		*Stable lines marking Rab proteins in zebrafish to visualize dynamics in early, late, and recycling endosomes, respectively, and endosome biology in vivo (Clark et al., 2011)*
*GalT-EGFP*		*Transgenic tool marking the specific trans-Golgi enzyme galactose-1-phosphate uridyl transferase (GalT) in zebrafish (Sepich and Solnica-Krezel, 2016)*
*Actin and microtubules (MT) cytoskeleton*		*Stable lines labeling intracellular actin (Riedl et al., 2008) and MT (Asakawa and Kawakami, 2010; Tran et al., 2012) for in vivo study of cytoskeleton dynamics in zebrafish*
*Lamp1/2*		*Transgenic tool labeling autolysosomes and other acidic compartments in zebrafish, useful to evaluate lysosome biogenesis dependent on the small GTPase regulation (Sasaki et al., 2017)*
*GFP-Map1Lc3*		*Stable line used to follow zebrafish autophagy in vivo (Moss et al., 2020)*

MitoID	RAB and other small GTPases	Molecular tool to identify effectors and regulators by *in vivo* proximity biotinylation approach with mitochondrially localized GTPases (Gillingham et al., 2019)

*Examples of specific tools used in zebrafish are italicized.*

#### RAS/MAPK Biosensors and Actuators

A variety of biosensors for the Ras/MAPK signaling are used *in vivo* (i.e., rodents, insects, and zebrafish; Hirata and Kiyokawa, 2019) to capture spatiotemporally cell dynamics, depending on ERK activity in the context of cell growth, differentiation, and migration. Classical fluorescence resonance energy transfer (FRET)–based extracellular-regulated kinase activity reporter (EKAR)–types biosensors exploit the ability of activated ERK to phosphorylate a substrate that triggers a conformational change of the sensor, such as to bring the FRET pair of fluorophores in close physical proximity (FRET pairs classically employing a GFP-like fluorescent protein as a donor and a red-emitting protein as an acceptor). This results in an increase of the FRET efficiency quantified by ratiometric measurements, in which the ratio between the intensities of the donor and acceptor fluorescence is calculated and correlates with the ERK activity (Harvey et al., 2008; Fritz et al., 2013). Alternatively, sensitive sensors have also been developed for fluorescent lifetime measurement (FLIM) quantification on the donor protein, which shortens as the FRET efficiency increases. Originally, Yasuda et al., 2006, established a 2-p (two photon microscopy)–FLIM–based RAS biosensor in which the FRET efficiency increased when active RAS-EGFP was recruited to the membrane to bind the RAS-binding domain of RAF-RFP. This tool was employed already to study the RAS signaling spreading dynamics for local long-term potentiation (LTP) in organotypic preparation of rodent hippocampal neurons. Moreover, FRET-FLIM ERK sensors (named EKA-Ret-cyto), which used a highly absorbent acceptor, were established and used in a deep 2-p confocal setting to capture ERK activity in subcellular compartments such as the dendritic spines. The sensor further contributed to clarify the role of Ras/MAPK signaling during LTP-dependent plasticity of hippocampal neuronal circuit (Tang and Yasuda, 2017).

Novel genetically encoded ERK sensors have been developed by innovative processes of dimerization-dependent fluorescent protein exchange, permitting a green-to-red shift in fluorescent intensity (Ding et al., 2015). On the other hand, sensitive kinase translocation reporters (KTRs) are able to convert the protein phosphorylation state into a nuclear–cytoplasmic shift in fluorescence that enables visualizing single-cell signaling dynamics of multiple events (e.g., to differentiate ERK and AKT activities downstream the RAS signaling, Maryu et al., 2016). In fact, these sensors are based on the ability of ERK to phosphorylate a substrate and mask, by conformational change, the nuclear localization signal, while unmasking the nuclear export signal. This results in an increase of cytoplasmic fluorescent upon ERK activation. Given that the fluorescent readout of KTR sensors depends on the shuttling between the nucleus and cytoplasm, it does not suffer from delays due to protein stability and expression level. The tool has been recently optimized for nematodes, to assess *in vivo* the dynamic function of Ras-dependent ERK activity in establishing cell competence and fate decision (de La Cova et al., 2017). The authors established a precise protocol for image analysis of ERK-nKTR in different developmental stages and contexts, including vulva morphogenesis, migratory muscle, sensory neurons, and gonad precursor cells. The work allowed mapping EGF-dependent modulation of frequency of ERK activity fluxes in real time with a specific spatial pattern in the different precursor cells, not possible with standard methods.

Similarly, a KTR dynamic reporter of ErK activity (DREKA) sensor has been established and successfully employed in zebrafish (Mayr et al., 2018). Dynamic Erk activity was registered in the dividing cells in the developing fish embryo during wound healing and for studying the uptake kinetics of chemical compounds influencing Ras/MAPK signaling relevant for preclinical applications. However, given that several factors might influence the readout based on the sensor nucleus–cytoplasm shuttling (i.e., nucleus morphology, expression levels of exportin proteins), resulting in inhomogeneous labeling of different cells, optimization of the DREKA sensor would be required for broader applications in the context of developmental dynamics and neuronal precursor cells.

Wong et al. (2018) have developed in parallel a stable transgenic fish expressing the FRET-based ERK biosensor Teen, proving already its usefulness to unravel ERK dynamics in a number of Ras/MAPK-controlled processes throughout developmental stages. A spatiotemporal map of Erk activity was generated in the entire developing organism from the early blastula until segmentation stages. Teen allowed discovering an overlooked domain of action of Ras/MAPK signaling during fish development in the caudal region of the neural tube, where the regulated activity of FGF-Erk, Wnt, and Bmp signaling contributes to shape the embryo axes and stem cell differentiation (neuronal and mesodermal fate). This zebrafish toolkit is of great potential to link precisely healthy and pathogenic molecular dynamics, involving Ras/MAPK signaling and cellular events *in vivo*, with considerable applications for understanding of RASopathies.

In parallel, other smart methods to visualize more dynamics are being developed (Ross et al., 2018), which will likely be translated *in vivo* in the future.

A deep understanding of signaling networks benefits also from genetically controlled actuators that can be used to perturb signaling and infer signaling rules. Tunable optogenetic control of Ras/MAPK activity was achieved employing optimized light-inducible dimers. In the OptoSOS system, the membrane-bound SsrA peptide of the α-helix of the plant-derived light-oxygen-voltage 2 (LOV2) domain is masked and cannot bind its receptor SsrB fused to a fluorescently tag SOS activator in the dark. The binding can be, however, triggered by blue light allowing strict spatiotemporal control of SOS membrane recruitment and thereby Ras/MAPK signaling activation. The use of this tool *in vivo* demonstrated the sensitivity of early *Drosophila* blastula and gastrula stages to ectopic Erk expression for axial morphogenesis (Johnson et al., 2017). Further investigations using OptoSOSO clarified the importance of a correct dosage and timing of the Ras/MAPK signaling for stem cell fate decision during these crucial developmental windows (Johnson and Toettcher, 2019). Furthermore, coupling precise optogenetic actuators of the Ras/MAPK signaling and quantitative reporting of ERK activity in a single cell line by CRISPR/Cas have allowed to describe the Ras/MAPK-dependent ERK pulses inducing immediate early gene transcription (Wilson et al., 2017).

Of note, S. Y. Shvartsman’s laboratory recently optimized a photoswitchable MEK device (psMEK) to manipulate the Ras/MAPK signaling using microscopy and Dronpa-based photo-dimerizable protein domains, already applicable to *Drosophila* and zebrafish (Patel et al., 2019). In the “off” state, the active site of constitutively active psMEK is blocked by Dronpa dimers. Upon illumination at 500 nm, the site is exposed via dissociation of the Dronpa dimers, and activation of Erk can occur (“on” state). Importantly, the signal can be reversibly switched off by using 400-nm light. The authors already used pMEK in *Drosophila* and zebrafish development and demonstrated that this actuator can be smartly utilized to classify mutations for an *in vivo* genotype–phenotype correlation analysis of RASopathies, based on the strength of signaling perturbation and known morphogenesis and organogenesis phenotypic readouts. Experiments in zebrafish already demonstrated the usefulness of this tool to probe the relevant time window in which the impact of the various *MEK* mutations causing gastrulation and axes defects influences other signaling networks that must be tightly controlled during the same processes (Patel et al., 2019).

#### RHO Biosensors and Actuators

Optimized versions of genetically encoded RHO sensors are also beingintensely utilized to observe RHO GTPases dynamics in various contexts since many years (Hodgson et al., 2010; Fritz et al., 2013; Donnelly et al., 2014; Boueid et al., 2020), including developments for cell-based high-throughput applications (Koraïchi et al., 2018). Classically, frogs were widely used to study RHO dynamics via simple fluorescent effector translocation probes and FRET biosensors. A first use involved the study of the modulation of Cdc42, Rac, and RhoA on proteoglycan and non-canonical Wnt signaling during NC migration *in vivo* (Matthews et al., 2008) and the observation of characteristic flares of RHO (dynamic accumulation of the active GTPases at the cell–cell junction) indispensable throughout embryo cell motility (Stephenson and Miller, 2017).

Ontogenetically induced actuators based on the LOV domains were engineered also to generate a photoactivable RAC1 by fusing the PHOT1 LOV2 to a constitutively active RAC1. Upon blue light illumination, the small GTPase is unmasked and able to constitutively bind its effectors and thereby trigger the downstream signal (Wu et al., 2009). By using the FRET sensor Raichu-Rac1, based on a CFP/YPF-mediated FRET upon the activation of the GTPase, combined with this type of actuator, it was possible to highlight the contribution of Rac1 in cell polarization and collective migration in *Drosophila* ovary development (Wang et al., 2010) and in neutrophil immune response of zebrafish (Yoo et al., 2010). Recently, a specific transgenic tool was generated to control optogenetically growth cone guidance mechanisms of growing motoneuron axons in zebrafish via the tissue-specific expression of PA-Rac1. The tool was employed to rescue innervation defects observed in mutant animals (Harris et al., 2020), demonstrating validity for study healthy and diseased neuronal circuit wiring.

Interestingly, genetically encoded in the GAL4/UAS versatile system, specific inhibitors of Cdc42 (*Myr-GFP-ACK42*) have been already used in combination to specific Cdc42 FRET sensor to assess the function of this small GTPases during angiogenesis (Wakayama et al., 2015).

Elegant functional studies in zebrafish exist, combining overexpression and knockdown approaches together with FRET sensors to model the dynamic activities of both Rac1 and RhoA in establishing actin-rich blebbing and retrograde actin flow for E-cadherin–dependent traction forces, respectively, in the context of early germ cells migration (Kardash et al., 2010). More recently, sophisticated deconvolution algorithms to obtain super-resolved images from PA-Rac1 experiments have allowed Zhang et al. (2019) to confirm a crucial role of Rac1 in mediating actin remodeling and filopodia stabilization for zebrafish pioneer axon formation of sensory neurons. Utility of FRET-based sensors is also clear from transgenic rodent models (Johnsson et al., 2014), for instance, to monitor RHO-dependent invasiveness of engrafted glioblastoma cells (Hirata et al., 2012). Noteworthy, an NIR FRET RAC1 biosensor for deep multiplexing imaging and signaling manipulation has been recently developed. The implementation of the most NIR FRET pair miRFP670–miRFP720 for this sensor enables the combinatorial use with classical CFP-YFP FRET pair for RHOA sensor upon optogenetic signal activation to study their concurrent dynamics during cell motility (Shcherbakova et al., 2018a).

In addition, exploiting the versatile GAL/UAS genetic system, Hanovice et al. (2016) established useful zebrafish GAL4-inducible transgenic lines for tissue and temporally tuned modulation and visualization of mutant (i.e., dominant negative) and wild-type Rac, RhoA, and Cdc42 (*10xuas:mCherry-F2A-myc-Rac*, *RhoA*, or *Cdc42*). The system is expandable to a range of mutants and allows systematic functional investigation of RHO protein–specific cells and developmental windows of interest and possible crosstalks in fish cancer models (Chew et al., 2014).

Specific transgenic lines to study the role of RHO proteins during immune response have also been developed (e.g., *mpeg:mcherry-2A-rac2*; Rosowski et al., 2016). Smart multiplexing transgenic tools in zebrafish permitted the discovery of Cdc42-induced “filopodia extensions” for mediating paracrine and large-range Wnt signaling in the context of zebrafish development, as discussed earlier (Stanganello et al., 2015).

Noteworthy, Kim et al. (2019) have established highly sensitive and expandable intensiometric biosensors for the simultaneous detection of smaller GTPases combined with optogenetic signaling actuation *in vivo*, previously used to describe the regulated activity of CDC42 and RAS in the context of rodent structural dendritic changes upon neurotrophic signaling activation. Furthermore, optogenetic activation of CDC42 was used to study the immune cell migration (O’Neill et al., 2016), which can be further exploited for counteracting the invasiveness of cancer cells (Palucka and Coussens, 2016).

### Vital Dyes and Transgenic Tools for Monitoring ARF and RAB-Regulated Intracellular Trafficking and Organelle’s Dynamics

At present, a series of tools are readily available in animal models to map intracellular trafficking events and investigate the role of small GTPase in these processes. Among vertebrate models, fish harbors good molecular devices not only for visualizing but also for manipulating organelle dynamics and cell biology of developing tissues in virtually any developmental stage.

The use of vital dyes and advanced imaging techniques is largely used to label intracellular compartments, molecules, and dynamics. For instance, *lifeact* allows actin network dynamic visualization via fluorescence recovery after photobleaching (Riedl et al., 2008). The rhodamine-labeled phalloidin, which selectively binds to F-actin, can be readily used to label actin filaments in zebrafish developmental studies (Li et al., 2008). Other dyes to label subcellular structures are widely employed to study organelles’ dynamics in disease models (e.g., lysosomes, Tseng et al., 2018). Besides these tools, all relevant intracellular organelles can be stably labeled by transgenic lines and/or transiently by libraries of constructs expressing fluorescently tagged markers for multiplexed imaging. In addition to standard subcellular markers labeling nuclei and cell membranes, specific organelles and vesicle markers are available in zebrafish (reviewed by Vacaru et al., 2014). Among those, GalT-GFP fish expressing galactose-1-phosphate uridyl transferase (Gerhart et al., 2012) are useful to map trans-Golgi, whereas Lamp2-mCherry can be used to visualize lysosomes (Sasaki et al., 2017), and GFP-Map1Lc3 has been already employed to image disease-associated autophagy *in vivo* (Moss et al., 2020).

A number of transgenic lines and constructs for labeling Rab and endosomal vesicles of different types (such as fluorescently labeled Rab5, Rab7, and Rab11 for early, late, and recycling endosomes, respectively) are successfully used for dynamic analysis of recycling endosomes *in vivo* (Clark et al., 2011), as well as transgenic fish lines marking actin, i.e., Tg (*uas:lifeact-GFP*) (Riedl et al., 2008) and a number of fish labeling MT such as the Tg (*UAS:EGFP-tuba2*) (Asakawa and Kawakami, 2010), the MT-associated doublecortin-like kinase Tg *(XlEef1a1:dclk2DeltaK-GFP*), the Eb3 + growing tip of the MT (Tran et al., 2012), and Tg (*bactin2:HsENSCONSIN17–282-3xEGFP*) fish expressing GFP-tagged MT-binding region of ensconsin (Wühr et al., 2011).

Notably, protein engineering translated into transgenic fish allows now to map with a substantial spatiotemporal resolution also highly complex phenomena, such as the site of neuronal protein synthesis during early zebrafish CNS development (Garcez Palha et al., 2018). More recently, Verweij et al. (2019) managed to develop a transgenic fish line expressing CD63-pHluorin for direct monitoring with high spatiotemporal accuracy of extracellular vesicles (EVs) dynamics secreted by multivesicular endosomes at an interorgan level. The work utilizes the *in vivo* whole-embryo reporter and demonstrated the dynamics of formation, transport, and function and the trophic role of the EV secreted by cells of yolk syncytial layer during development. In addition, the FRET type of RAB biosensors also exists and has been developed and used to monitor, for instance, Rab5 activity in phagosome maturation of immune cells (Kitano et al., 2008). Of note, Gillingham et al. (2019) have reported the development of *MitoID*, an innovative methodology for identifying a wide range of small GTPases’ effectors and regulators employing *in vivo* proximity biotinylation of mitochondrial-restricted GTPases and found several RAB effectors. Lastly, via advanced protein design and chemical strategies, Conformational sensors for GTPase activity (COSGAs) awaiting *in vivo* applications, allowing direct observation of GTPase activation state, were recently used to detect RAB1 and K-RAS activity *in vitro* and quantification of RAB1 GTP-/GDP ratio at high spatial and temporal resolution (Voss et al., 2016).

The development of optogenetic devices to manipulate intracellular trafficking has been notoriously more challenging. Nevertheless, plant photoreceptors responsive to UV light have been genetically engineered for controlling protein secretion and used to investigate dendritic cargo secretion (Chen et al., 2013). More recently light-induced actuators to perturb RAB-dependent intracellular trafficking have also been established, which permit fast, tunable, and reversible interference of membrane dynamics, protein sorting, and endosomes signaling. In the IM-LARIAT system engineered by Nguyen et al. (2016), a subdomain of the blue light–sensitive cryptochrome 2 (CRY2) from *Arabidopsis thaliana* self-oligomerizes immediately together with CIB1 fused to RAB upon blue light illumination. This results in aggregation and perturbation of the GTPases’ activity during intracellular trafficking at various levels, depending on the targeted RAB protein (early, late, or recycling endosomes, ER–GA transport, and secretion). The tool was already implemented in rodent hippocampal neurons to study the contribution of RAB5 and RAB11 in influencing dendritic growth’s rate.

## Conclusion

Next-generation sequencing of previously unrecognized pediatric conditions disclosed an unforeseen impact of small GTPases of the RAS superfamily on the pathogenicity of a growing number of developmental disorders. The precision of this approach is bringing up amazing possibilities for investigating unexplored mechanistic principles of developmental biology, re-employing classical animal models. Continuous advances in the field of high-resolution microscopy, genetic engineering, and synthetic biology for optimized biosensors and actuators for *in vivo* studies are now unfolding, well exemplified by the zebrafish model. We anticipate that the expansion and optimization of these tools for multiplexing *in vivo* signal visualization and manipulations will have an unprecedented impact for the spatiotemporal investigation of developmental signaling networks modulated by small GTPases in health and disease. In conclusion, an integrated pipeline from patients back to precise organismal biology in the global context of embryo development represents the blueprint for a modern global health care response to the burden of the ever-increasing pediatric genetic diseases, critical for developing tailored measures in the rapidly emerging field of precision medicine.

## Author Contributions

AL, GF, MT, and BD wrote the manuscript. AL conceived and generated all the illustrations. AL and GF conceived the table. GF and MV generated the table. All authors contributed to the article and approved the submitted version.

## Conflict of Interest

The authors declare that the research was conducted in the absence of any commercial or financial relationships that could be construed as a potential conflict of interest.
